# Integrating literature-constrained and data-driven inference of signalling networks

**DOI:** 10.1093/bioinformatics/bts363

**Published:** 2012-06-25

**Authors:** Federica Eduati, Javier De Las Rivas, Barbara Di Camillo, Gianna Toffolo, Julio Saez-Rodriguez

**Affiliations:** ^1^Department of Information Engineering, University of Padova, Padova, 31050, Italy, ^2^European Bioinformatics Institute (EMBL-EBI), Wellcome Trust Genome Campus, Cambridge, CB10 1SD, UK and ^3^Bioinformatics & Functional Genomics Group, Cancer Research Center (CSIC/USAL), Salamanca, E37007, Spain

## Abstract

**Motivation:** Recent developments in experimental methods facilitate increasingly larger signal transduction datasets. Two main approaches can be taken to derive a mathematical model from these data: training a network (obtained, e.g., from literature) to the data, or inferring the network from the data alone. Purely data-driven methods scale up poorly and have limited interpretability, whereas literature-constrained methods cannot deal with incomplete networks.

**Results:** We present an efficient approach, implemented in the R package CNORfeeder, to integrate literature-constrained and data-driven methods to infer signalling networks from perturbation experiments. Our method extends a given network with links derived from the data via various inference methods, and uses information on physical interactions of proteins to guide and validate the integration of links. We apply CNORfeeder to a network of growth and inflammatory signalling. We obtain a model with superior data fit in the human liver cancer HepG2 and propose potential missing pathways.

**Availability:** CNORfeeder is in the process of being submitted to Bioconductor and in the meantime available at www.cellnopt.org.

**Contact:** saezrodriguez@ebi.ac.uk

**Supplementary information:** Supplementary data are available at *Bioinformatics* online.

## 1 INTRODUCTION

Information about signalling networks is increasingly abundant. Thanks to novel high-throughput methods, large amounts of data about the interactions among proteins is available, which is encompassed in (unsigned and undirected) protein–protein interaction networks (PINs) (Pieroni *et al.* 2008). More precise (but with less coverage) information is derived from literature and is often described by means of signed and directed causal interactions among proteins. These give rise to what we will call here prior knowledge networks (PKNs). PKNs are partially collected in different databases [e.g. KEGG (Ogata *et al.* 1999), Reactome (Joshi-Tope *et al.* 2005), WikiPathways (Pico *et al.* 2008) and several are accessible via the portal Pathway Commons (Cerami *et al.* 2011)]. These databases typically contain literature-derived interactions curated with different degrees of stringency, and based on experimental publications under different experimental conditions using different cell types.

PKNs are, for example, very useful to study topological properties of networks (Ma'ayan *et al.*, 2005) or to map data (Ideker and Sharan 2008, Terfve and Saez-Rodriguez 2012). However, they are not functional in the sense that they cannot be used for simulation of a signalling process and therefore prediction of the outcome of a certain experiment, which is fundamental to understand signal transduction and its alterations.

The most common way to model a signalling network is to write down its biochemistry and subsequently translate it to a mathematical form, typically a system of differential equations (Aldridge *et al.*, 2006). However, information in PKNs often lacks the required mechanistic detail. In these cases, logic formalisms are a useful approach since all they need is to add logic gates to the existing (signed and directed) interactions.

One can generate logic gates by manual curation based on literature, for example (Calzone *et al.*, 2010, Saadatpour *et al.*, 2011, Samaga *et al.*, 2009), reviewed in (Morris *et al.*, 2010, Watterson *et al.*, 2008). An alternative to manual curation consists of generating a logic model from the PKN that is subsequently trained to experimental data (Saez-Rodriguez *et al.*, 2009). This method, implemented in the Bioconductor package CellNOptR (http://www.ebi.ac.uk/saezrodriguez/software.html), provides context-specific models with predictive power. It is efficient at handling large amounts of data as the space of possible models is limited by the prior knowledge. This key feature of the approach, however, is also its main limitation: there might be missing links as databases are not complete, and the effect of cross talk between pathways is often not taken into account in the canonical linear representation of the pathways. Hence, adding links to the PKN based on the dedicated data can lead to an improved goodness of fit (Saez-Rodriguez *et al.*, 2009).

With a different and complementary perspective, different ‘reverse engineering’ methods have been used to infer networks from perturbation experiments using data-driven methods that do not rely on prior knowledge of the network (Bansal *et al.*, 2007, Markowetz, 2010). Most of these methods were first developed for transcriptional data but can be applied also to signalling data. For example, in (Ciaccio *et al.*, 2010) Bayesian networks (Pe'er, 2005) were used to infer the connections between 67 proteins with high-throughput data collected using a micro-western array. Two mutual information-based approaches, the ‘algorithm for the reconstruction of accurate cellular networks’ (ARACNe) (Margolin *et al.*, 2006) and the ‘context likelihood of relatedness’ (CLR) (Faith *et al.*, 2007), were also applied to the same dataset to corroborate the results. Different methods were also applied in the context of the DREAM initiative (www.the-dream-project.org) for the DREAM4 Predictive Signalling Network Challenge (Prill *et al.*, 2011). Twelve research groups inferred signalling networks from perturbation experiments data and were evaluated based on the accuracy of their predictions of the outcome of the network under different experimental conditions. One of the methods that performed best in this task was a simple approach, strictly data-driven, that encodes significant effects of stimuli and inhibitors on measured proteins in a cause–effect network (Eduati *et al.*, 2010).

These purely data-driven methods need to consider all possible topologies, and thus in general, need more data and scale-up worse than methods that rely on a given topology such as CellNOptR. Furthermore, the resulting data-driven networks (that we will call here DDNs) are limited to interactions between perturbed and measured nodes that are only a subset of the nodes involved in the pathways. Thus, DDNs are not as biologically interpretable as the PKNs and mapping DDNs to PKNs is not simple as one link in the inferred network can generally correspond to multiple links in the PKN. Hence, it is not trivial how to correctly map this relationship.

In this article we attempt to combine the strengths of literature-based and data-driven inference methods. We describe a procedure (implemented in the R package CNORfeeder), to integrate prior knowledge encoded in the PKN with data-driven information obtained using reverse-engineering approaches. PINs are used to prioritize links and to provide experimental support for them, and thus help to discriminate among options and add information on integrated links. The resulting network is then trained against experimental data to obtain a final refined model that has a better fit to data with respect to the PKN, highlighting plausible links that were missing in the PKN. We illustrate its application with a signalling network encompassing multiple pathways and readouts trained with data from the liver cancer cell HepG2. We show how CNORfeeder provides a significantly improved fit based on links supported by known interactions among proteins.

## 2 METHODS

**Fig. 1.**
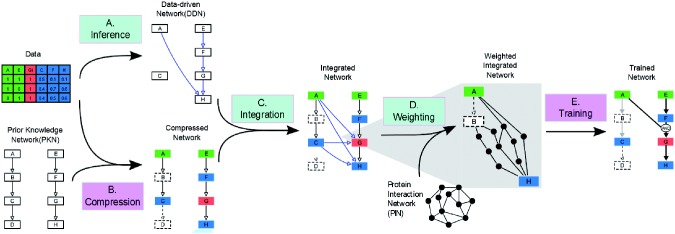
Integrated pipeline of CNORfeeder (light boxes) and CellNOptR (dark boxes). (**A**) Data are used to infer, using reverse-engineering methods, a strictly DDN; (**B)** the PKN is compressed according to the data (dark, middle and light grey nodes are, respectively, stimulated, inhibited and measured), removing non-identifiable nodes (dashed); (**C**) the compressed network is integrated with the DDN (dotted links are obtained from the DDN and continuous links from the PKN); (**D**) information derived from PINs are used to support and prioritize integrated links; (**E**) The integrated network is used as input for the training: in the trained model, thick black lines denote interactions (and gates) in the trained model, and light-grey links denote presence in the integrated network but not in the trained model

We implemented CNORfeeder, an R package designed to be integrated with methods based on prior knowledge such as CellNOptR as shown in Figure 1. The integrated pipeline can be summarized in the following steps:

- *Inference (CNORfeeder)*. A strictly DDN is inferred from available data using different reverse-engineering methods (so far FEED, Bayesian networks, ARACNe and CLR). This network is specific for the experiments under study, thus it only includes perturbed and measured nodes and does not exploit information available in literature.
- *Compression (CellNOptR)*. The PKN is compressed according to the procedure detailed in (Saez-Rodriguez *et al.*, 2009). First, if a node has no readout downstream of it (such as D in Fig. 1), its state cannot be inferred (it is non-observable) and is not considered. Similarly, if a node has no perturbation upstream, it is not included as it will not be affected. Then, nodes that are neither perturbed nor measured are bypassed so that their compression does not change the logic of the remaining nodes (e.g. B which is between A and C in Fig. 1).
- *Integration (CNORfeeder)*. The compressed network is expanded using the DDN in order to include links that are missing in the a priori information but that seem to be supported by data.
- *Weighting (CNORfeeder)*. PINs are used to support and prioritize the integrated links.
- *Training (CellNOptR)*. The integrated network is finally converted into a superstructure containing all possible logic gates compatible with the network. If a node (such as G) is affected by multiple nodes (A and F), then both an OR and an AND gate are created. Then a genetic algorithm is used to search for the model contained in the superstructure which best describes the data [as determined by a score based on the mean squared error (MSE)] with the minimum number of links. The objective function is modified with respect to that introduced in (Saez-Rodriguez *et al.*, 2009) to include additional penalization for the integrated links using weights derived from PINs [Equation (2)].

Steps performed by CNORfeeder will be detailed in the following sections.

### 2.1 Inference using reverse-engineering methods

CNORfeeder can in principle leverage any network inference method. So far, we have integrated the following:

*FEED inference* is the R implementation of an improved version of the algorithm described in (Eduati *et al.*, 2010). The inference of the network can be divided in two steps. Fist, perturbation experiments are used to infer a Boolean table for each measured protein, codifying if a particular stimulus inhibitor combination affects the protein. A stimulus or an inhibitor significantly affects an output protein if it is able to modify its activity level by a quantity that exceeds the uncertainty associated with its measurement. These Boolean tables are than translated into links among stimulated, inhibited and measured nodes, giving rise to the inferred network (see Supplementary Material for more details).

*Bayesian Network inference* There are different approaches to derive causal influences between measured proteins using Bayesian networks. We have used the ‘catnet’ R package (available from http://cran.r-project.org/web/packages/catnet/index.html) to derive categorical Bayesian networks from static data (see Supplementary Material for more details).

*Mutual information networks* This class of methods computes the mutual information matrix between the measurements associated with different proteins and, based on that, infers an undirected network. In particular, ARACNe and CLR algorithms as implemented in the ‘minet’ R package (Meyer *et al.*, 2008) (see Supplementary Material for more details), are included in CNORfeeder.

**Fig. 2.**
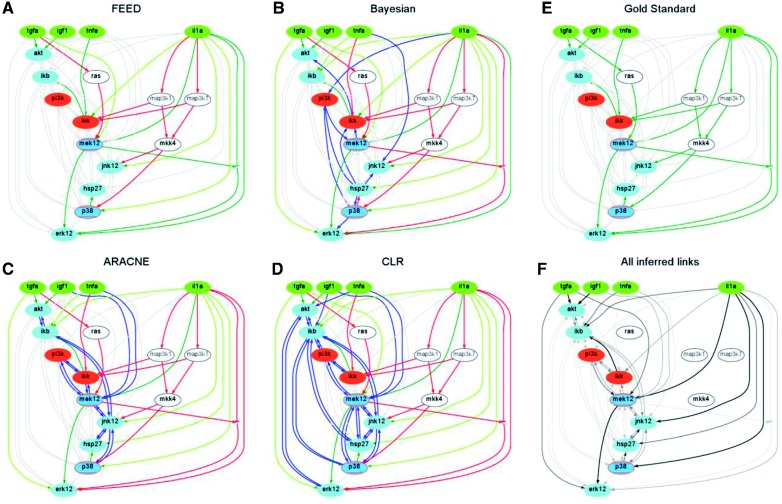
Reverse engineering of a Gold Standard network (**E**) using four different inference methods (**A–D**). Dark, middle and light grey nodes are, respectively, stimulated, inhibited and measured. Link styles represent the comparison of the inferred networks (DDNs) with the Gold Standard: dark thick continuous for links in both networks, dark thin continuous for links in the DDN that correspond to a path in the Gold Standard, dashed for links in the Gold Standard not present in the DDN and dotted for links in the DDN that are not in the Gold Standard, light grey for links that are not in the network under examination but are in one of the other networks. In panel (**F**) the gradation of grey represent the consistency between DDNs in panels (A–D)

*In silico* data were generated using a ‘Gold Standard’ or true network, depicted in Figure 2E, to compare the four algorithms. The ‘Gold Standard’ was randomly generated and interpreted as a logic Boolean model to simulate perturbation experiments using CellNOptR. This was performed by stimulating (nodes in dark grey), inhibiting (nodes in middle grey) and measuring (nodes in light grey) the specified proteins. These *in silico* data were then given as input to the inference methods; resulting networks (DDNs) are shown in Figure 2A–D. The advantage of this approach, with respect to the use of real data, is that the Gold Standard can be used to compare the performances of the different methods.

In Figure 2A–D dark grey links are those that are perfectly reconstructed being present both in the Gold Standard and in the inferred network. Some of the links in the Gold Standard are not inferred by the algorithm (dashed ones), for example, *tgfa→ras* or *ras→mek12* for FEED. However in some cases the algorithm is still able to infer at least an indirect link (light grey ones), for example, *tgfa→mek12* for FEED. Dotted links are those that are inferred by the algorithm but do not correspond to links in the Gold Standard even as indirect links. In this example, FEED is able to infer all links in the Gold standard, at least as indirect ones, without including any false positive links. It is important to notice that mutual information approaches do not allow for determining the directionality of the links; for light and dark grey links the directionality was assessed based on comparison with the Gold Standard to simplify the figure. A similar approach can be used in real cases by comparison with the PKN, but there is no way to assess the directionality of missing links. In Figure 2F, links are represented with different gradations of grey according to the consistency between the analyzed inference methods: black links are reconstructed by all methods. As expected, links involving proteins that are neither perturbed nor measured (white nodes) cannot be reconstructed by any inference algorithm. However, those nodes can be important for the signalling network and often there is available literature-derived information about their role. This is one of the reasons why it is fundamental to integrate the information derived from data-driven inference methods with the prior knowledge obtained from other resources.

### 2.2 Integration with the PKN

Some of the links included in the DDN might be missing in the PKN, and are thus candidates to be integrated with it. However, the PKN generally includes more nodes with respect to the DDN and a link in the DDN could, in some cases, correspond to more than one link in the PKN. As shown in Figure 1C, if there is a connection between a cue (i.e. a stimulated or an inhibited protein) and a measured protein in the DDN (e.g. from A to H), we have to connect all nodes in the different paths corresponding to that link. This means adding a link not only from the cue to the measured protein, but also from all nodes downstream of the cue, until the following cue is reached, to all nodes upstream of the measured proteins, until the previous measured protein is reached.

### 2.3 Protein–protein interaction network

The human PIN was built using a unified PPI dataset obtained as APID (Prieto and De Las Rivas, 2006), by the combination of interactions coming from six source databases. The starting whole dataset was composed of 68 488 human physical PINs validated by at least one experimental method and reported in one article published in PubMed. From this dataset we obtained two PPI subsets with increasing confidence: a set of 28 971 interactions validated by at least one ‘binary’ experimental method [binary as defined in (De Las Rivas and Fontanillo, 2010)]; a set 6033 interactions validated by at least two experimental methods, one of them binary.

### 2.4 Weighting and training of integrated network

The integrated network is then optimized using CellNOptR to find the model which best describes the data using information from PINs to differently prioritize integrated links. As described in (Saez-Rodriguez *et al.*, 2009), a bipartite objective function is used to balance fit and size, that is, to find models with good fit to the data but with the minimum number of links. Defining *P* as a Boolean vector encoding the candidate solution model (value 1 or 0 is assigned depending if the link is included or not in the model), the function that is minimized during the optimization process is the following:
(1)
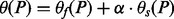

where 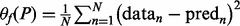 is the MSE deviation between the normalized experimental data (continuous values between 0 and 1), and the model prediction (binary values 0 or 1), for all *N* measured data points. 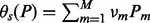 is a term to penalize increasing model size according to a tunable parameter 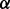. The size penalty 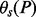 is computed as the weighted sum of the *M* links, which are mathematically hyperedges in the hypergraph that defines the model; see (Saez-Rodriguez *et al.*, 2009) for details. The weight (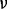) is given by the number of starting nodes, for example, hyperedge A AND B*→*C is weighted twice compared with A*→*C. In Equation (1) it is possible to include a tunable parameter 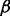 to allow a stronger penalization of links integrated to the PKN leading to
(2)
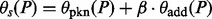

where the size penalty 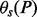 is the sum of two terms: one for the links in the PKN (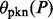) and one for integrated links (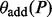). This is motivated by the fact that, being supported by literature, links in the PKN are more reliable with respect to links integrated using data-driven approaches and they should be prioritized in the training.

Additionally, integrated links can be differently prioritized based on information derived from PINs: the basic idea is that if, for a directed link A*→*B integrated in the PKN, there is a corresponding path in the PIN, it is more plausible that there is a molecular pathway A*→*B. Because shorter paths are more feasible, as a first approximation the shortest path length between A and B in the PIN can be used as a reliability score for the integrated link. Since the optimization is performed on a compressed version of the PKN, one link integrated in the compressed network generally corresponds to multiple possible links integrated in the PKN (Fig. 1E). Thus, the reliability score for each integrated link *i* is given by 
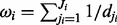
, where *j_i_* = 1, … , *J_i_* are the links in the PKN corresponding to the integrated link *i* in the compressed network. The shortest path *d* is computed using the Dijkstra’s algorithm implemented in the igraph R package (Csardi and Nepusz, 2006) considering the PIN as a graph where the weight of the edges is the inverse of the number of experiments (experimental evidences) that validate it.

Thus, the penalty for all A integrated links into the compressed network *P*, can be defined as
(3)
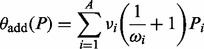



The training step, to find the *P* that minimizes 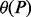 in Equation (1), is performed with CellNOptR using a genetic algorithm that explores the *P*-space. The genetic algorithm is run multiple times, and in each run the values for the explored models is recorded, so that at the end a family of models is reported.

## 3 RESULTS

The method was applied to a dataset of a human liver cell line (HepG2) from the DREAM 4 challenge (Prill *et al.*, 2011), where the phosphorylation of seven proteins (akt, erk12, ikb, jnk12, p38, hsp27, mek12) is measured 30 min after combinatorial stimulation with four ligands (tnfa, il1a, igf1, tgfa) and four inhibitors (pi3k, ikk, p38, mek12). The level of phosphorylation of proteins is measured using the Luminex xMAP assay and provides a value of the phosphorylation in arbitrary units, that can be used to compare values at two conditions. In our case we compare the values between 0 and 30, and this change is a proxy of the induced activation of the corresponding protein. The normalization of this data to a value between 0 and 1 is achieved using a method based on a set of thresholds as described in (Saez-Rodriguez *et al.*, 2009). According to the CellNOptR pipeline, the PKN was first compressed removing all non-observable and non-controllable nodes and then expanded as described in (Saez-Rodriguez *et al.*, 2009) to include all possible combinations of AND and OR gates compatible with the network obtaining a total of 62 hyperedges. Additionally, 18 links inferred using FEED were integrated in the network according to the procedure previously described and the integrated network was used for optimization using CellNOptR.

**Fig. 3.**
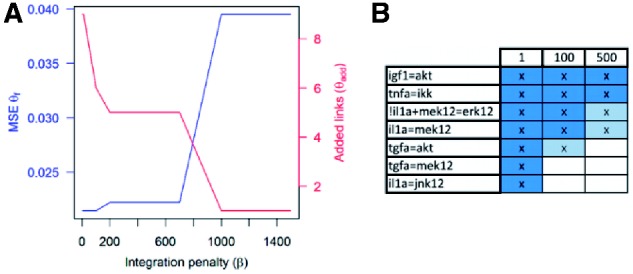
(**A**) Effect of tuning parameter *β* on the number of integrated links (continuous line) and on the fit (MSE, dashed line). (**B**) Links integrated for different values of *β* (1, 100, 500); a reduced number of links is selected when using PIN to prioritize links (dark grey)

**Fig. 4.**
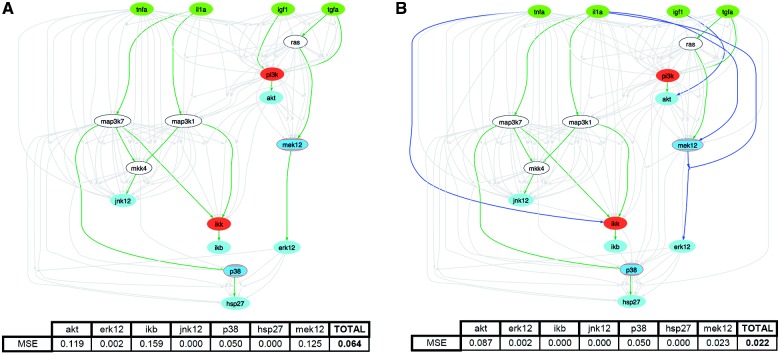
Results of the training of the compressed model (**A**) and of the integrated network (**B**) against data using CellNOptR. Dark, middle and light grey nodes are, Respectively, stimulated, inhibited and measured. Selected links are represented with continuous line if derived from the PKN and dotted line if integrated, links not selected are in light grey. In the tables the fit (in terms of MSE) is reported for each measured protein along with the sum for all proteins

Fixing *α* = 0.001, the influence of the integration penalty (*β*) on the number of integrated links selected by the optimization process on the fit of the optimal model to the data (in terms of MSE) was tested as shown in Figure 3. As expected, a low value of *β* obtains the best fit but at the price of a high number of integrated links included in the optimal model (9 with *β* = 1). An increase of the value of *β* decreases the number of selected integrated links but worsens the fit to the data. With *β* = 1000 only the integrated link tnfa*→*ikk is included in the optimal model: the presence of this link is well supported by the data since it lowers the MSE from 0.064 (the optimal fit obtained with CellNOptR using as input the non-integrated network) to 0.040. The integrated links can be ranked as shown in Figure 3B according to the highest value of *β* allowing their selection and thus according to their effect on the improvement of the fit. A lower number of links is selected when using the PIN to additionally penalize unsupported links (highlighted in dark grey in Fig. 3B). Those links, combined with the information from the PIN, suggest possible missing connections in the PKN. For example, in the PIN there is an interaction between the adaptor irs1 and the kinase pdk that would justify the link igf1*→*akt in the compressed network since, in the PKN (Fig. S2 in Supplementary Material), igf1 binds to its receptor and pdk regulates akt (links igf1*→*igfr and pdk1*→*akt in Supplementary Fig. S2; note that in Fig. 4 the compressed networks are shown and thus intermediates igfr, irs1s and pdk1 are not present). Therefore the path igf1*→*igfr *→*irs1s*→*pdk1*→*akt is supported by a combination of literature and interaction data. Similarly, to support the link tnfa*→*ikk there is a validated interaction between the tnfa receptor and cot, a protein that activate ikk, leading to the combined pathway tnf*→*tnfr*→*cot→ikk.

In Figure 4A and B the results of CellNOptR optimization (with *β* = 700) are shown using as input the compressed network and the integrated network, respectively. In the upper panels, optimal models are shown: links selected by the optimization algorithm are represented with continuous line if derived from the PKN and dotted line if integrated using FEED. In the lower panel the improvement in the fit is shown (from 0.064 to 0.022), which is particularly large for proteins ikb, mek12 and akt. In this case study, using the same parameter setting (*α* = 0.001, *β* = 700), networks integrated using ARACNe and CLR do not provide an improvement of the fit, whereas Bayesian networks obtain an MSE of 0.040 (see Supplementary Material). As for the computational times, FEED, CLR and ARACNe inferred the network in ∼1 second whereas Bayesian inference took ∼1 h on a cluster.

To evaluate the scalability of our method, we applied CNORfeeder to a larger dataset obtained also in the cell lines HepG2, comprising 7 stimuli, 7 inhibitors and 15 readouts (Saez-Rodriguez *et al.*, 2009). We obtained comparably good results (see Supplementary Material).

Furthermore, we investigated the ability of our method to capture feedback loops, which are fundamental in the regulation of signal transduction. We constructed a toy model containing a negative feedback loops and simulated data at two different time points (10 and 30 min). We used FEED as a reverse-engineering method to retrieve, from the data, a link of the feedback that was missing in the PKN and then applied a recently implemented package of CellNOptR (www.cellnopt.org) that, looking also at the second time point, was able to select all links of the feedback loop (see Supplementary Material for further details).

## 4 DISCUSSION

In this article, we present an approach that integrates literature-constrained and data-driven methods to efficiently infer signalling networks from experimental data collected under perturbation experiments with different stimuli and inhibitors. The procedure is implemented in the R package CNORfeeder and consists of (i) inference of a data-derived network (DDN) using strictly data-driven reverse-engineering methods (so far FEED, Bayesian networks and mutual information approaches); (ii) integration of the DDN with a literature-derived PKN, using PINs to prioritize and validate integrated links; and (iii) training of the integrated network against data using CellNOptR to obtain a logic model that best describe the data with the minimum number of links.

**Fig. 5.**
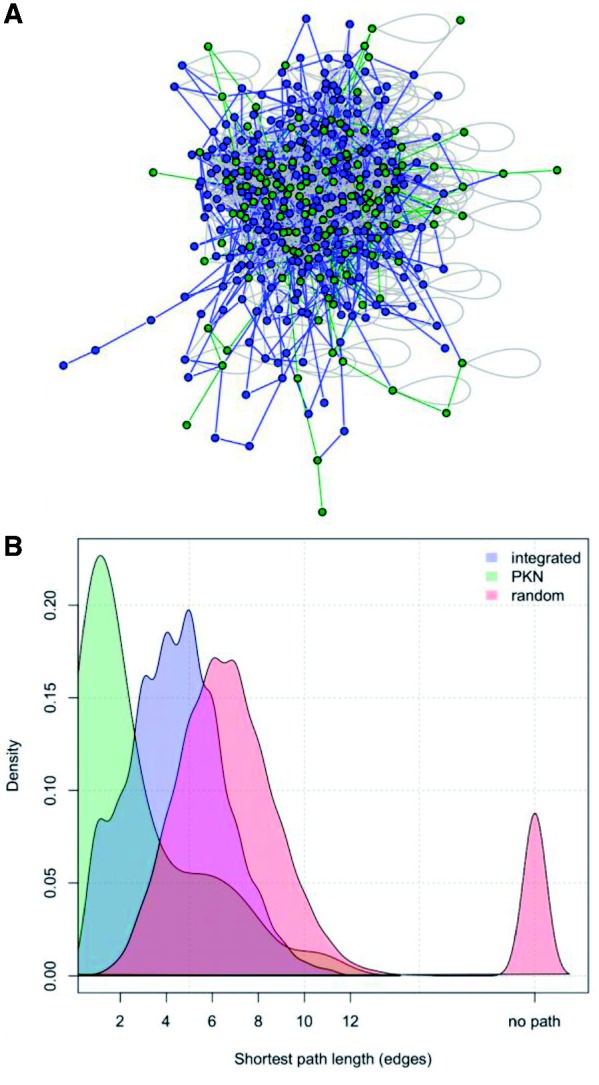
Mapping of the PKN to the PIN. (**A**) Represents the subgraph of the PIN that include only nodes belonging to the PKN (dark grey) and nodes used in the mapping of integrated links (light grey); the network was plotted with R package igraph. The same colour code is used for the edges: as expected, shortest paths between nodes in the PKN (dark grey) are generally shorter than paths used to map integrated links (light grey). This is highlighted also in (**B**) where the density of the shortest path length (in terms of number of edges) is plotted for integrated links, for links in the PKN and for random links

Links that improve the fit to data with respect to the PKN alone may be missing due to the difficulty assembling all available pathway information or because of incomplete knowledge of the biology. PINs are used as a complementary source of information to tackle this problem. PINs contain physical interactions between proteins, including those that potentially lead to protein activations, and they typically include more nodes and many more links than those based on literature-derived pathways. For this reason they have been proposed to extend pathways (Glaab *et al.*, 2010) but they have the main limitation of a lack of directionality. PINs are also known to have high false positive and false negative rates, and we, therefore, used a highly curated PIN that integrates different sources and experimental techniques. This PIN seems to be quite complete for the pathways we studied (canonical pro-growth and inflammatory pathways) since we verified that for links in the PKN there is, generally, also a direct connection in the PIN (Fig. 5). Interestingly, when mapping to the PIN the links integrated in the PKN, we found a corresponding short path that does not pass through other nodes of the PKN. To limit the effect of false positive links in the PIN when searching for the shortest path, we weighted the edges according to the number of experimental evidences that support them. The length of the shortest path is then used to differently prioritize the integrated links in the training of the network, but other metrics could be used to discriminate between links. PINs were previously shown to be potentially useful to find previously unknown modulators of signalling pathways in (Vinayagam *et al.*, 2011), where a Bayesian learning strategy was applied to assign directionality to a comprehensive PIN exploiting information on the shortest path from membrane receptors to transcription factors. In our method, we can take advantage of the directed links inferred via reverse engineering to limit the paths present in the PIN we integrate, and limiting the search space for the optimization algorithm.

We have used different data-driven inference methods, and applied them to both *in silico* (to reverse engineer a benchmark network with known topology) and real data (to integrate links missing in the PKN that improve the fit of the model to the data in the liver cancer cell line HepG2). Each reverse-engineering method has specific features and can be suitable for different needs: for example, Bayesian networks can provide statistically rigorous results but at the price of high computational costs, whereas mutual information approaches are computationally fast but are limited mostly by the lack of directionality of the inferred links. FEED seems to be particularly suitable to infer causal networks from single-stimulus/single-inhibitor experiments with low computational costs but, as for now, does not exploit data from all multiple combinatorial perturbation experiments.

It is not the purpose of this study to compare reverse-engineering methods (which would require a larger set of benchmark networks with known topology and a more realistic simulation of experimental data). The spirit of the article is more in line with the lesson derived from the DREAM challenges (Marbach *et al.*, 2010; Prill, 2011) that different approaches can provide complementary insights into the same problem. We have thus employed various approaches and we plan to extend it to others in the future. Furthermore, some reverse-engineering methods can use prior knowledge, in particular Bayesian inference methods (Bender *et al.*, 2011, Mukherjee and Speed, 2008), so that we could use the PKN or results from the training with CellNOptR to guide a further search for novel links.

To conclude, the integration of literature-constrained and data-driven inference methods overcomes the limitations of both: for purely data-driven inference methods, the poor scalability (as the search space increases exponentially) and limited biological interpretability (since they are limited to measured and perturbed proteins excluding intermediate ones), and for methods constrained to prior knowledge their inability to overcome incompleteness in the networks. We propose here an approach (and software package) to combine them that is effective and extendable to include other methods.

## Supplementary Material

Supplementary Data
