# Supplementary material for: Integrating literature-constrained and data-driven inference of signalling networks
Source: Bioinformatics. 2012 Jun 25;28(18):2311–7. doi: 10.1093/bioinformatics/bts363 (PMC3436796; doi:10.1093/bioinformatics/bts363)
Supplement: Supplementary Data [file supp_28_18_2311__index.html]

Integrating literature-constrained and data-driven inference of signalling networks — Supplementary Data 

## Supplementary Data

files

**Files in this Data Supplement:**

- Supplementary Data - pdf file
