# Supplementary material for: Integrating literature-constrained and data-driven inference of signalling networks
Source: Bioinformatics. 2012 Jun 25;28(18):2311–7. doi: 10.1093/bioinformatics/bts363 (PMC3436796; doi:10.1093/bioinformatics/bts363)
Supplement: Supplementary Data [file supp_bts363_SupplementaryMLSB.pdf]

Category

Integrating literature-constrained and data-driven inference of signalling networks (Supplementary)

Federica Eduati[1,2], Javier De Las Rivas[3], Barbara Di Camillo[1], Gianna Toffolo[1] and Julio Saez-Rodriguez[2,*]

[1]Department of Information Engineering, University of Padova, Padova, 31050, Italy

[2]European Bioinformatics Institute (EMBL-EBI), Wellcome Trust Genome Campus, Cambridge, CB10 1SD, UK

[3]Bioinformatics & Functional Genomics Group, Cancer Research Center (CSIC/USAL), Salamanca, Spain

Received on XXXXX; revised on XXXXX; accepted on XXXXX

Associate Editor: XXXXXXX

1 REVERSE ENGINEERING METHODS

1.1 FEED inference

In this manuscript we use an improved version of the method presented in (Eduati,F., et al. 2010). As in the original implementation, the inference of the network using FEED is performed in 2 steps: 1. A Boolean table is built for each protein, having a column for each stimulus and a row for each inhibitor and codifying in each cell if the stimulus/inhibitor combination significantly affects the protein ([a,b]: a is 1 if the stimulus affects the protein, 0 otherwise and b is the same but for the effect of the inhibitor); 2. a cause-effect network is reconstructed from the Boolean tables adding links between stimuli, inhibited and measured proteins according to effects codified in the tables.

The first improvement with regard to the previously published method of the method is the inference also of negative links: the significance of the effect, for example, of a stimulus is now assessed based on its ability to alter (increase or decrease) the activity level of the protein of a quantity that exceeds the quantity associated with the measurement thus introducing the absolute value in the formula

$$|test - ref| > k \times s \qquad (S1)$$

where, in this example, *test* is the activity level of the protein in presence of the stimulus, *ref* is the basal level, s is the associated measurement error and *k* is a proportionality constant. The sign of the regulation is encoded in another table with the same structure. The same approach is used also to analyse the effect of the inhibitors.

The second improvement is the use of Boolean tables to reconstruct the network that allows considering information derived from the tables in the integration of the prior-knowledge network (PKN). For example, in Figure S1 A it is shown how *igf1* and *tgfa* affect *akt* and this is represented with the corresponding links in the reconstructed network. The table also tells us that this effect is not mediated by any of the inhibited proteins, and this is taken into account in the integration process. The example of Figure S1 C shows how information encoded in different tables can be combined in the inference of the network: both *il1* and *tgfa* affect *mek12* that, in turn, affects *erk12*. However, *il1a* has no effect on *erk12* and this is represented with a negative AND gate.

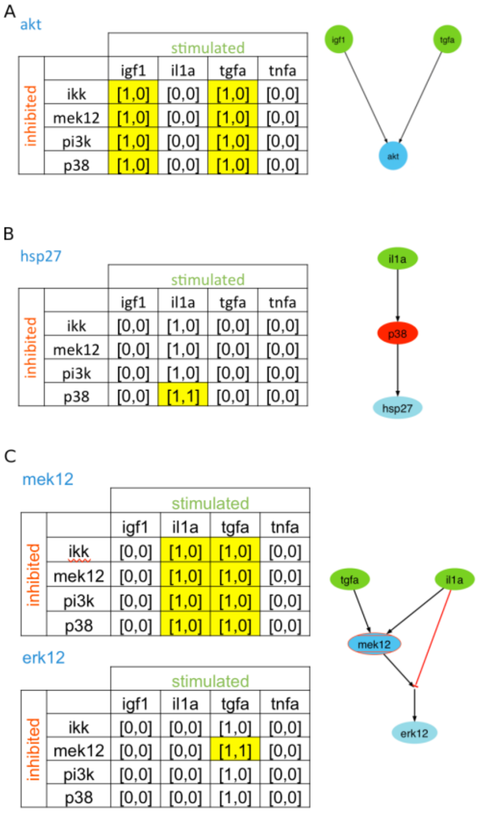

Fig. S1. Reconstruction of cause-effect network from Boolean tables using FEED. For each measured protein, a Boolean table is inferred from data and encodes the effect of stimuli (columns, first value of each cell) and inhibitors (rows, second value of each cell).

© Oxford University Press 2005

1.2 Bayesian network inference

The 'catnet' R package (available from http://cran.r-project.org/web/packages/catnet/index.html) allows the inference of categorical Bayesian networks based on Maximum Likelihood estimation. Data used in paragraph 2.6 of the manuscript (already normalized between 0 and 1 as described in (Saez-Rodriguez,J., et al. 2009)) were divided in two categories using 0.5 as threshold. The maximum number of parents for each node was set to 3 for all nodes except for the ones representing stimuli that are expected to have no parents. The cnSearchSA function was used to search in the space of node orders by Simulated Annealing setting parameters in order to allow the algorithm to run long enough to have a stable solution. This function gives as output a list of networks with different complexities that describe data reasonably well. Instead of considering only one of this networks, we derived a consensus model including all links with frequency > 0.1.

1.3 Mutual information networks

The 'minet' R package (Meyer,P.E., et al. 2008) (available in Bioconductor) implements different mutual information network inference methods. The fist common step is the computation of the mutual information matrix that is then given as input to the different methods that differently compute the edge score for each pair of nodes. We focused in particular on ARACNe and CLR to infer network but the same package also allows the use of other mutual information based approaches (relevance network, MRNET). Since mutual information approaches do not allow for determining the directionality of the links, both directions are considered. In the CNORfeeder package we allow to give as input a network to be used as comparison to assess the directionality of some links. This network can be a benchmark network as in the paragraph 2.1 of the manuscript, or the prior-knowledge network (PKN) in the real case.

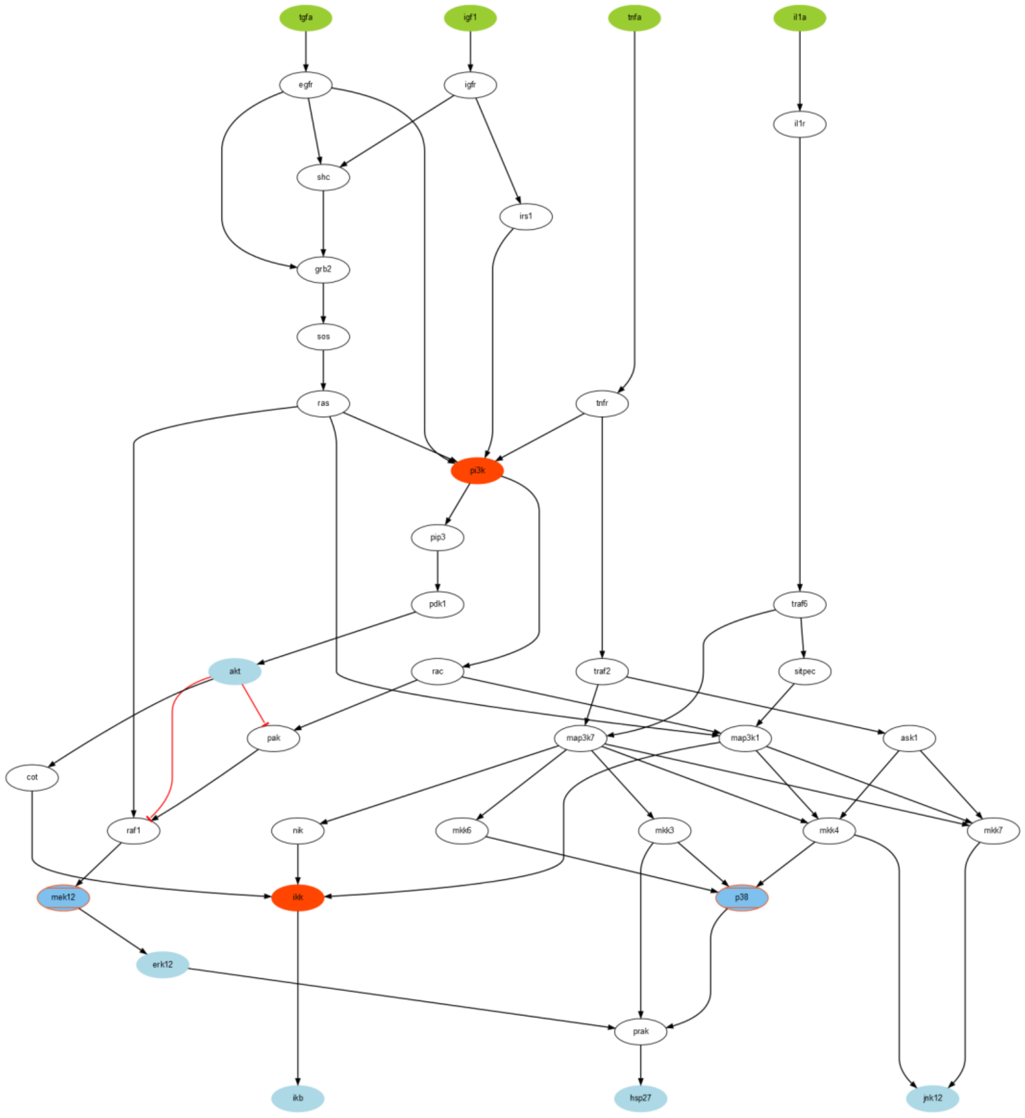

Fig. S2. Prior Knowledge Network (PKN) of growth and inflammatory signalling pathway.

2 APPLICATION TO GROWTH AND INFLAMMATORY SIGNALLING IN LIVER CANCER CELL LINE HEPG2

The PKN of growth and inflammatory signalling is shown in Fig S2. Results of the application of Bayesian networks and Mutual Information based approaches, discussed in Results section of the manuscript, are shown in Figure S3.

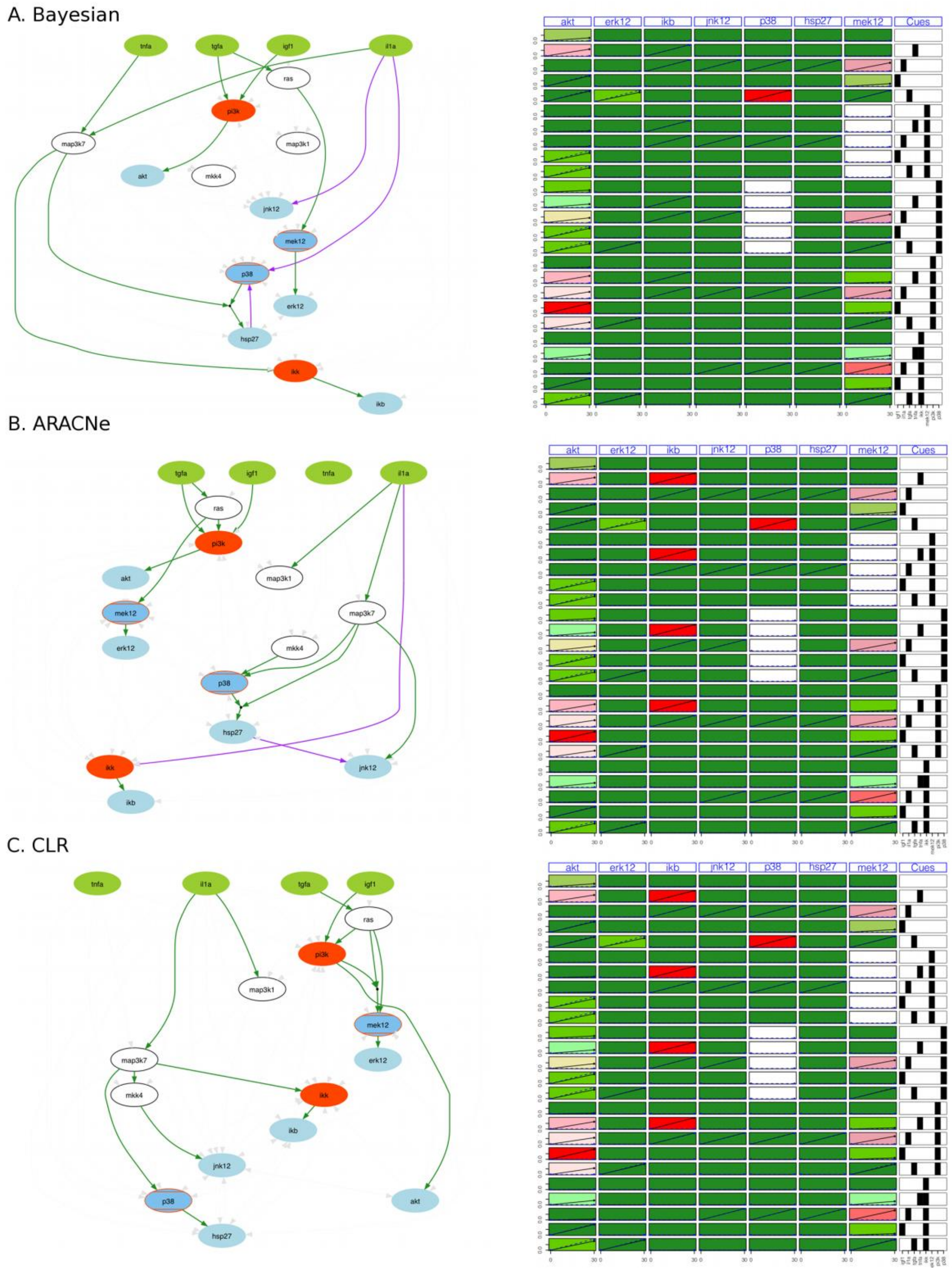

Fig. S3. Results of the training against data using CellNOptR the network against data using CellNOptR using Bayesian network inference (A), ARACNe (B) and CLR (C). Left panels: selected links are represented in green if derived from the PKN and blue if integrated. Right panels: the consistency between model predictions and data are shown highlighting in green good fit and in red bad fit. Simulated values are in dashed-blue lines and experimental data in black. Each column corresponds to a readout and each row to an experiment.

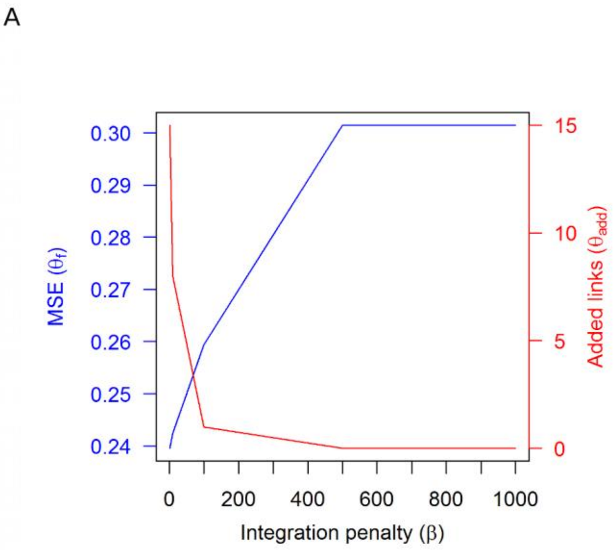

B

	1	10	100	500	1000
TNFa=IKK	x	x	x		
IL1a=IKK	x	x			
IL1a=JNK12	x	x			
IL1a=p38	x	x			
IL1a=cJUN	x	x			
IL1a=IRS1s	x	x			
TGFa=IRS1s	x	x			
cJUN=p70S6	x				
cJUN=mTOR	x				
p53=p70S6	x				
IL1a=p70S6	x	x			
IL1a=MEK12	x				
TGFa=MEK12	x	x			
p53=CREB	x				
TGFa=CREB	x				
p53=mTOR		x			
JNK12=P70S6	x				
JNK12=MTOR	x				
JNK12=CREB	x				
CJUN=CREB	x				
IL1a=CREB		x			

Fig. S4. Panel A: effect of tuning parameter β on the number of integrated links and on the fit (MSE) for the large dataset. Panel B: links integrated for different values of β (1, 10, 100); Links in light blue and purple are selected only using the unweighted and weighted approach respectively while links in dark blue are selected in both cases.

3 SCALABILITY OF THE METHOD

CNORfeeder was testes also on a larger dataset with 7 stimuli, 7 inhibitors and 15 readouts used in (Saez-Rodriguez,J., et al. 2009). We inferred the data-driven network (DDN) using FEED and than integrated it with the PKN from (Morris,M.K., et al. 2011) (a modified version of the (Saez-Rodriguez,J., et al. 2009) one) according to the pipeline. Using the integrated network as input for the training to data using CellNOptR (setting α=0.01 and β=10 and equal weight for all integrated links) permits to obtain an improved fit (MSE=0.24 Figure S5 B) with respect to the use of the compressed network derived only from a priori information (MSE=0.30 Figure S5 A). In Figure S4 A the effect of the increase of the value of β on the fit (MSE) and on the number of integrated links selected during the training is shown. In Figure S4 B, integrated links selected for different values of β are listed and highlighted in dark blue if they are selected both using PINs to prioritize links (weighted) and giving the same weight to all integrated links (unweigthed). Links in light blue and purple are selected only using the unweighted and weighted approach respectively. Weighting the links permits to discriminate between links that has the same effect on the improvement of the fit: for example, considering results for β=10, both *il1a* → *p70s6k* and *p53* → *p70s6k* leads to a better fit of *p70s6k* data when stimulated by *il1a* but the first one is suggested by information derived from the PIN.

4 FEEDBACK-LOOPS

Feedbacks loops are often playing an important role in signalling network; in the toy example in Figure S6 we show how the proposed approach is able to unreaveal also this tipical pattern of signalling networks. In this example, in silico data (shown in lower panel of Figure S6) were simulated at 2 different time points (10 and 30 minutes) and the procedure described in the paper was then applied, using FEED as reverse-engineering method, to infer the network using recently implemented package of the platform CellNOptR that takes into considerations both time points (see www.cellnopt.org). Results are shown in the upper panel of Figure S6: looking at the feedback between SOS, RAF1 and ERK, the link *RAF* →*ERK* (in blue), that was missing in the PKN, was inferred using FEED and selected during the training to complete the feedback loop. The negative link *ERK* →*SOS* (light green) can be selected by CellNOptR only when looking also at the second time point (30 min) because it is needed to explain the decrease in RAF1 and ERK activity following the increase at time 10 min.

REFERENCES

Eduati,F., et al. (2010) A Boolean approach to linear prediction for signaling network modeling. *PLoS One*, 5, e12789.
Meyer,P.E., et al. (2008) minet: A R/Bioconductor package for inferring large transcriptional networks using mutual information. *BMC Bioinformatics*, 9, 461.
Morris,M.K., et al. (2011) Training signaling pathway maps to biochemical data with constrained fuzzy logic: quantitative analysis of liver cell responses to inflammatory stimuli. *PLoS Comput.Biol.*, 7, e1001099.
Saez-Rodriguez,J., et al. (2009) Discrete logic modelling as a means to link protein signalling networks with functional analysis of mammalian signal transduction. *Mol.Syst.Biol.*, 5, 331.

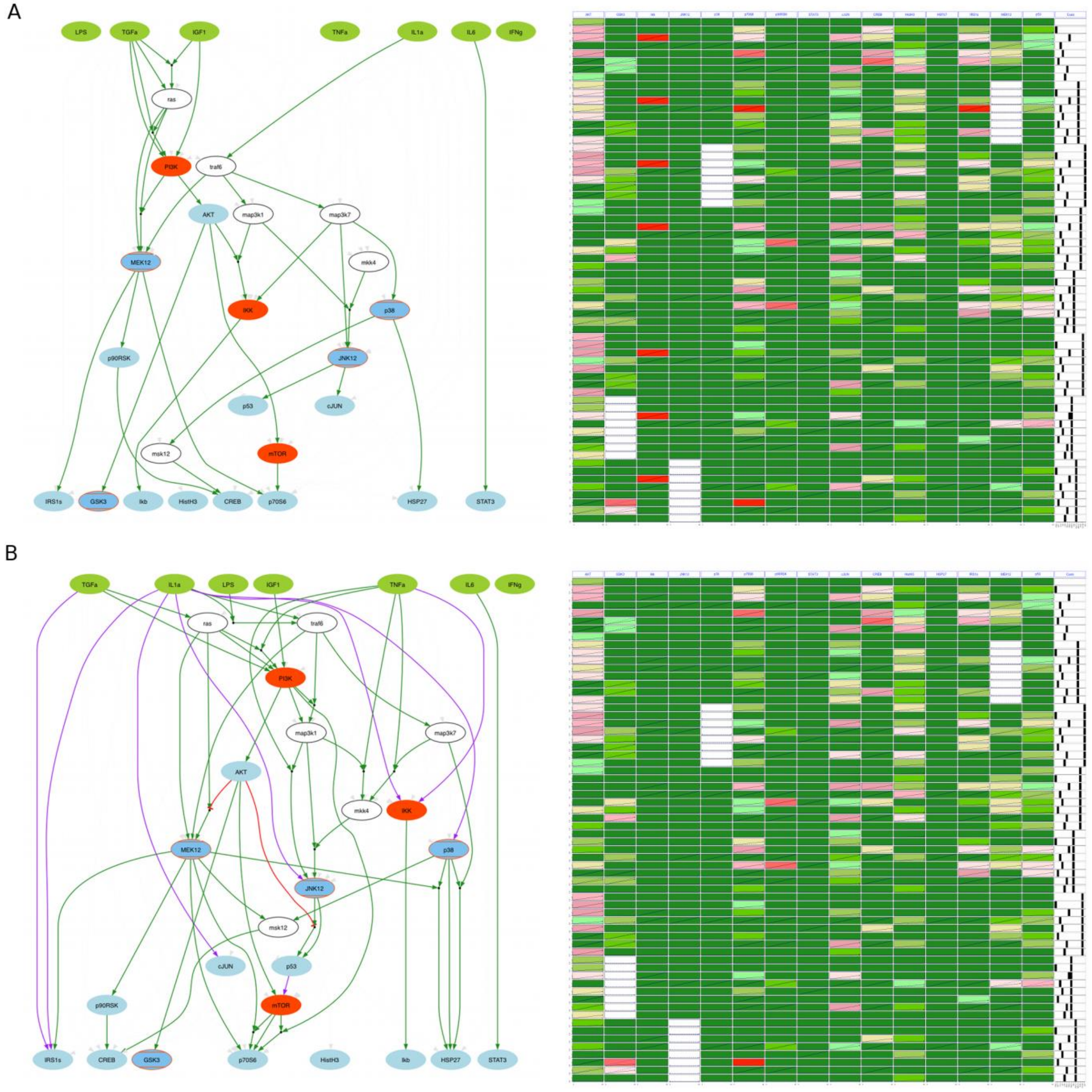

Fig. S5. Results of the training of the compressed model (A) and of the integrated network (B) against data using CellNOptR, for the large dataset. Left panels: selected links are represented in green if derived from the PKN and blue if integrated. Right panels: the consistency between model predictions and data are shown highlighting in green good fit and in red bad fit. Simulated values are in dashed-blue lines and experimental data in black. Each column corresponds to one readout and each row to an experiment.

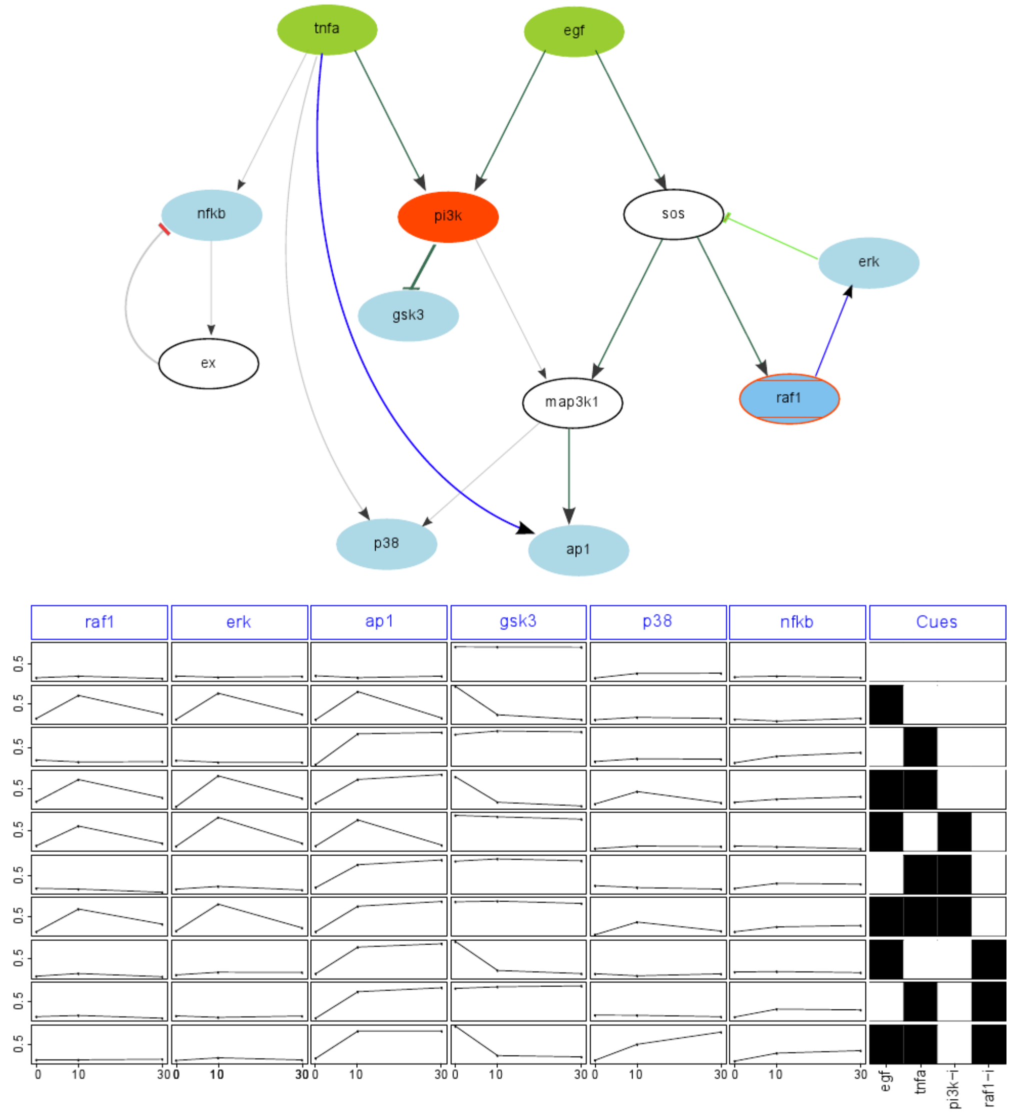

Fig. S6. Toy model with feedback loops. The model in the upper panel is the result of training to the data in the lower panel. Green, red and blue nodes are respectively stimulated, inhibited and measured. Selected links are represented in green if derived from the PKN (dark green for the first time point, light green for the second) and blue if integrated, links not selected are in grey.
